# Indicators for evaluating public speaking in adults: a scoping review

**DOI:** 10.1590/2317-1782/e20250234en

**Published:** 2026-04-27

**Authors:** Willian Hote Scanferla, Anna Carolina Ferreira Marinho, Adriane Mesquita de Medeiros, Leticia Caldas Teixeira

**Affiliations:** 1 Programa de Pós-graduação em Ciências Fonoaudiológicas, Universidade Federal de Minas Gerais – UFMG - Belo Horizonte (MG), Brasil.; 2 Departamento de Fonoaudiologia, Faculdade de Medicina, Universidade Federal de Minas Gerais – UFMG - Belo Horizonte (MG), Brasil.

**Keywords:** Communication, Public Speaking, Verbal Communication, Nonverbal Communication, Voice

## Abstract

**Purpose:**

To map the indicators used to observe adults’ public speaking and to identify the forms of measurement.

**Research strategies:**

The acronym PCC (Population, Concept, and Context) and combined descriptors were used to search the LILACS (BVS), MEDLINE (PubMed), Scopus (Elsevier), Web of Science (Clarivate), and Cochrane databases.

**Selection criteria:**

Studies on the assessment of adult public speaking, conducted through observation by evaluators/judges, published in English, Portuguese, or Spanish, with no restrictions regarding year of publication or methodological design, were included. Studies involving individuals with stuttering or neurological disorders, those employing technological or non-human assessment, those focused on foreign language teaching, or those based solely on self-perception were excluded.

**Results:**

A total of 35 studies published between 2002 and 2024 were included, with a concentration in 2016 and a predominance of articles from the Scopus and Web of Science databases. Most of the identified indicators were developed by the authors themselves or adapted from existing protocols. They were distributed across major domains: discourse structure, language use, supporting materials, vocal expressiveness, and nonverbal behaviors. The most commonly used measurement scale was the Likert-type scale.

**Conclusion:**

The mapping identified recurring indicators in public speaking assessment: discourse structure (introduction, development, organization, clarity, conclusion), supporting resources, language (audience appropriateness, argumentation, pronunciation, fluency), nonverbal behaviors (eye contact, gestures, posture, facial expressiveness), and vocal expressiveness (volume, rhythm, pitch, articulation, modulation). The predominant form of measurement was the Likert-type scale.

## INTRODUCTION

Public speaking is defined as the ability to deliver structured and intentional oral communication in front of a group to inform, influence, or entertain the audience^(1,2)^. This challenging skill is fundamental in various professional and academic fields and is considered a differentiator for personal and professional success, meeting the demands of the job market, which seeks qualified and multifunctional professionals^(2-4)^. Due to its importance, many people dedicate themselves to improving it^(1,2,4)^.

Public speaking is known to use verbal resources (including the message, choice of words, and preparation of speech), nonverbal resources (including body language and supporting visual signs), and vocal resources (including vocal dynamics, such as pitch, loudness, intonation, accentuation, modulation, pauses, rhythm, and speech speed)^(3,5,6)^. Other dimensions also influence the effectiveness of the presentation, such as interaction with the audience, the speaker's confidence, and the overall impact of the speech, and are frequently analyzed in evaluations of communicative performance^(2,4,5)^.

Studies on oral communication training frequently select indicators for judges to evaluate communicative aspects before and after intervention or mentoring^(3,7,8)^. However, there is no consensus in the scientific literature regarding the selection of these observable indicators, whether in validated instruments or in protocols without formal validation. Authors point out that the lack of uniformity highlights the diversity of criteria adopted by researchers and reinforces the need for greater efforts in constructing common parameters that favor practice and comparability between studies, contributing to the consolidation of the field^(2,8,9)^.

Given this gap and considering exclusively evaluators/judges’ observation assessment, it is relevant to identify the indicators and measurement methods they use, aiming to offer an updated overview that supports evaluation and enables the analysis of communicative skills in public speaking.

In this context, scoping reviews play a fundamental role in mapping the available evidence, identifying research gaps, and guiding new studies and practices^(10,11)^. This approach makes it possible to recognize the most convergent indicators among studies for evaluating the skills that need improvement in speakers in future public speaking assessments^(2,12)^.

Hence, this study aimed to map the indicators used to observe adults' public speaking and to identify the measurement methods.

## METHODS

### Research strategy

This scoping review followed the Joanna Briggs Institute (JBI) guidelines for scoping reviews^(11)^, as described in the PRISMA Extension for Scoping Reviews (PRISMA-ScR). The protocol for this scoping review was registered in the Open Science Framework (doi:10.17605/OSF.IO/7VKR4).

The study was designed with the acronym PCC (P – population, C – concept, and C – context), as follows: P – adults; C – public speaking assessment by speech-language-hearing pathologists and communication instructors; and C – public speaking and studies conducted in clinical or training settings. The research question was, “What indicators are used to observe the public speaking of adults, how are they measured, and what convergences stand out among them?”.

### Search

The search was conducted electronically on the LILACS (BVS), MEDLINE (PubMed), Scopus (Elsevier), Web of Science (Clarivate), and Cochrane databases. The search strategies were developed based on uniterms indexed on Medical Subject Headings (MeSH), Health Sciences Descriptors (DeCS), and free terms related to PCC. They were adapted for each database, using varied combinations and the Boolean operators AND and OR to obtain the final strategies. Table 1 presents the initial search strategy used in the databases.

**Table 1:** Initial search strategy in databases

**Databases**	**Strategy**
LILACS (BVS)	(tw: "Comunicação Verbal" OR "Discurso Público" OR "Fala Pública" OR "Falar em Público" OR oratória OR "Public Speaking" OR "Hablar en Público" OR oratoria OR "Discurso Público" OR "Hablar en Público" OR "Comunicação não Verbal" OR "Nonverbal Communication" OR "Comunicación no Verbal") AND ("Instrumento de Avaliação" OR "Escala de Avaliação" OR avaliação OR escala OR instrumentos OR indicadores OR "Assessment Instrument" OR "Assessment Scale" OR evaluation OR assessment OR scale OR instruments OR indicators OR "Instrumento de Evaluación" OR "Escala de evaluación" OR evaluación) AND ( db:("LILACS"))
PubMed/Medline	(("Public Speaking" OR "Verbal Communication" OR "Non-Verbal Communication")) AND (("Assessment Instrument"[Title/Abstract] OR "Assessment Scale"[Title/Abstract] OR Evaluation[Title/Abstract] OR Assessment[Title/Abstract] OR Scale[Title/Abstract] OR Instruments[Title/Abstract] OR Indicators[Title/Abstract]))
Scopus (Elsevier)	("Public Speaking" OR Oratory) AND ("Assessment Instrument" OR "Assessment Scale" OR Evaluation OR Assessment OR Scale OR Instruments OR Indicators)
Web of Science (Clarivate)	("Public Speaking" OR "Verbal Communication" OR "Non-Verbal Communication") AND ("Assessment Instrument" OR "Assessment Scale" OR Evaluation OR Assessment OR Scale OR Instruments OR Indicators)
Cochrane	("Public Speaking" OR "Verbal Communication" OR "Non-Verbal Communication") AND ("Assessment Instrument" OR "Assessment Scale" OR Evaluation OR Assessment OR Scale OR Instruments OR Indicators)

### Selection criteria

Two reviewers conducted the data search, selection, and extraction stages independently; a third reviewer was responsible for resolving any disagreements. The searches were carried out between July and September 2024. They were restricted to indexed journals and did not include gray literature, such as theses, dissertations, papers presented at events, and institutional documents.

The review included studies on evaluators/judges’ observation assessment of public speaking in adults, published in English, Portuguese, or Spanish, without restriction as to the year of publication or methodological design. The language restriction aimed to ensure feasible analysis and the team’s accurate interpretation of the findings.

Studies involving individuals with stuttering or neurological disorders, using technological or non-human assessment, focusing on foreign language teaching, or based solely on self-perception were excluded. The latter was intentional, as this scoping review aimed to map indicators observed and judged by evaluators/judges.

### Data analysis

Studies were selected in two stages. The article titles, abstracts, and keywords were initially analyzed for inclusion; then, the full texts were read to apply the exclusion criteria. This process took place between August and September 2024, using the online platform Rayyan QCRI^(13)^ for reference management and removal of duplicate studies.

The extracted data encompassed the studies’ specific details of the PCC and the main findings on the parameters used in assessing public speaking in adults. They included database, title, authors, year of publication, language, study objective, evaluation method (author-developed or validated questionnaire, with instrument identification), indicators used in public speaking analysis, response scale (Likert-type scale, numerical ordinal, or descriptive ordinal; qualitative or quantitative), and validity evidence. The extracted results were organized in a previously prepared Excel spreadsheet to facilitate data analysis and synthesis.

## RESULTS

**Figure 1:**
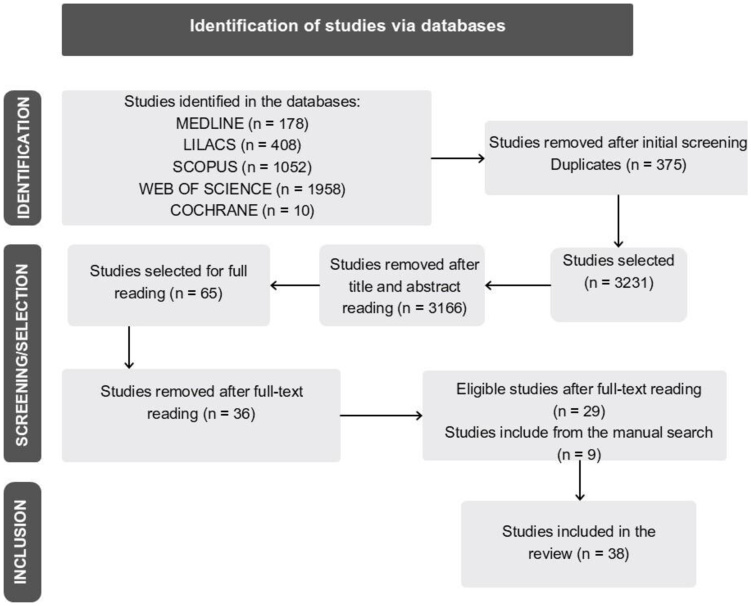
Flowchart of the search and selection of studies for inclusion in the research.

The initial search yielded 3,606 articles, identified in different databases, notably Scopus and Web of Science, in addition to manual searches. After applying the eligibility criteria, 26 studies were selected for analysis. There was no disagreement between the two reviewers responsible for the screening, thus eliminating the need for a third reviewer. Nine studies identified through manual searches of references of the selected articles were also included, totaling 35 studies in this review (Figure 1). The final articles were published between 2002 and 2024, with the highest concentration in 2016, when five studies were published.

**Caption:** n = number of studies

Among the indicators identified, the majority (26; 74.3%) were developed by the authors, while nine (25.7%) corresponded to validated protocols.

Table 2 shows that the most used protocol in four studies was the eight-item Competent Speaker Speech Evaluation Form (CSSEF), with a descriptive ordinal response scale and one open-ended question. The holistic version of the CSSEF has two items with both ordinal and descriptive response scales and was used in three studies. The Public Speaking Competency Instrument (PSCI) has 20 items, a Likert-type response scale, and was used in two studies. The Public Speaking Competence Rubric (PSCR) has 11 items, a Likert-type response scale, and was used in two studies. The *Escala de Evaluación de la Competencia Oral Componente No Verbal* (ECO-CNV; Spanish for Oral Competence Assessment Scale – Nonverbal Component) was found in one study in this review, has five items, and its measurement is done using a numerical ordinal scale. The protocols present a diversity of indicators, some grouped by major domains, others evaluated in isolation. Most instruments assess the discourse structure, considering the introduction, body, organization, and conclusion, with specific subdivisions that include clarity and objectivity. The instruments often assess supporting resources, although with different nomenclatures. Only one instrument did not include this indicator.

**Table 2:** Matrix for analyzing validated public speaking instruments

**Instrument**	**No. of items**	**Domains**	**Indicators**	**Studies**
PSCI	20	Introduction	Ability to attract attention. Clear purpose. The introduction is identifiable.	^(4,14)^
		Body	Key points and organization of the speech. Supporting material.	
		Conclusion	The conclusion and its purpose are identified. Closing points. Strong conclusion.	
		Delivery	Speech rate. Voice volume. Gestures. Eye contact. Body posture. Vocal and body expressiveness.	
		Overall Competency	Descriptive	
PSCR	11	There are no domains; the items/indicators are evaluated.	Topic appropriate for the audience. An introduction that guides the audience. Organized speech. Convincing supporting material. Reinforced conclusion. Word choice. Vocal expressiveness (volume, tone, rhythm). Nonverbal behaviors (posture, gestures, expressions, eye contact); adaptation to the audience. Skillful use of visual aids. Argumentation (persuasion, evidence, credibility).	^(15,16)^
ECO-CNV	5	Nonverbal communication	Body language; Facial expression; Eye contact; Flow; Volume	^(17)^
CSSEF	8	Public speaking skills	Topic selection and delimitation. Thesis clarity. Supporting material. Organizational standards. Appropriate language. Vocal variety (rhythm, tone, intensity); Pronunciation/grammar/articulation. Physical behaviors (posture, gestures, movements, expressions, eye contact, clothing). General comments.	^(18-21)^
Holistic CSSEF	2	Preparation and content; Presentation and delivery	Topic selection/definition. Communication of purpose. Supporting material. Organizational standards. Appropriate language. Vocal variety. Pronunciation/articulation. Nonverbal physical behaviors. General comments.	^(18,19,21)^

**Caption:** PSCI = Public Speaking Competency Instrument; PSCR = Public Speaking Competence Rubric; ECO-CNV *= Evaluación de la Competencia Oral Componente No Verbal*; CSSEF = The Competent Speaker Speech Evaluation Form; CSSEF-Holistic = The Competent Speaker Holistic Speech Evaluation Form

The instruments assess language with at least one indicator, encompassing aspects such as careful word choice, adaptation to the audience, argumentation (such as persuasion or credibility), good pronunciation, and fluency of speech (such as pauses and interjections).

All instruments include nonverbal behaviors, such as gestures, eye contact, body posture, body expressiveness, and facial expressions.

Most instruments analyzed include indicators related to vocal expressiveness, especially word articulation, speech rate, volume, tone of voice, vocal plasticity, and speech rhythm.

In two instruments, the evaluator can record a qualitative observation at the end of the evaluation, while only one includes a quantitative judgment of overall competence.

Table 3 shows a description of the indicators from the 26 studies that used non-validated instruments, with their respective response categories. Indicators focused recurrently on structural aspects of discourse, such as introduction (n = 3; 11.5%), organization (n = 10; 38.5%), clarity of ideas (n = 4; 15.4%), and conclusion (n = 1; 3.8%). Regarding nonverbal behaviors, eye contact (n = 17; 65.4%), gestures (n = 14; 53.8%), body posture (n = 11; 42.3%), and facial expressions (n = 8; 30.8%) stood out. In the language domain, six studies addressed language appropriateness to the audience (n = 6; 23.1%). In the domain of vocal expressiveness, the most frequent indicators were volume (n = 14; 53.8%), rhythm (n = 4; 15.4%), tone (n = 8; 30.8%), and articulation (n = 8; 30.8%). Other observed aspects included the quality of the theme (n = 1; 3.8%) and the use of supporting materials (n = 5; 19.2%). The instruments use different response scale formats (Table 4), including Likert-type scales (n = 11; 42.3%), numerical scales (n = 8; 30.8%), descriptive ordinal scales (n = 5; 19.2%), yes/no scales (n = 5; 19.2%), and visual analog scales (n = 2; 7.7%), with simple scores or weighted averages. Some instruments allow raters to record qualitative (n = 7; 26.9%) and quantitative (n = 2; 7.7%) observations at the end of the assessment.

**Table 3:** Matrix for analyzing non-validated public speaking instruments

**INDICATOR**	**STUDIES**
Overall assessment	^(12,22-28)^
Persuasion	^(7,12,29,30)^
Confidence	^(8,12,22-24,26-28,30,31)^
Emotion	^(8,29)^
Interaction	^(7,12,23,29,30,32)^
Spontaneity/Delivery	^(3,27,33)^
Discourse organization	^(3,7,8,22-24,28,29,34,35)^
Introduction	^(7,29,35)^
Clarity of ideas	^(26,27,29,35)^
Body of discourse/Content of speech	^(33,35-37)^
Conclusion	^(29)^
Quality of the topic	^(29)^
Language	^(7,23,29,31,32,36)^
Interjections	^(7,8,22,24,26-29,31,34,38,39)^
Anxiety	^(3)^
Fluid presentation	^(8,36)^
Negative content	^(34)^
Argumentation	^(23,27,32,35)^
Vocal psychodynamics	^(40)^
Vocal expressiveness	^(29)^
Vocal quality	^(8,41)^
Resonance	^(8,25,42)^
Tone of voice/pitch	^(3,8,25,27,30,41-43)^
Speech rate	^(3,8,25,34,37,40,41)^
Pause	^(8,23-25,27,31,34,37,40,42,43)^
Melody/melodic curve	^(25,37,40)^
Intonation	^(7,22,24,26,28,29,31,32,35,36,38-40)^
Rhythm	^(7,29,32,35)^
Intensity/Volume/Loudness	^(3,7,8,23,25,27,29,34,37-39,41-43)^
Articulation	^(8,23,25,30,37,40-42)^
Voice projection	^(35)^
Acoustic analysis	^(42)^
Emphasis	^(8,25,37,40)^
Gestures	^(3,8,22-29,33,35,37,38,43)^
Body Expressiveness	^(8,22,24,26,28,33,34,37-39,43)^
Facial expressions	^(3,8,25,27,29,35,39,43)^
Body posture	^(7,22-27,29,34,35,43)^
Eye contact	^(7,8,22-24,26,28,29,31,32,34-39,43)^
Head movements	^(25,33)^
Supporting materials	^(7,23,27,29,32,35,36)^
Presentation time	^(7,31,32,35)^
Clothing	^(23,36,37)^

**Table 4:** Description of the response categories and analysis methods of the validated and non-validated instruments identified in the studies

**Validated instruments**	**Scale type/Response categories**	**Analysis method/Score**
PSCI^(4,14)^	Likert scale (5 points, Excellent=5 to Poor=1)	Simple summation (20–100 points)
PSCR^(15,16)^	Likert scale (5 points) (Poor = 0 to Advanced = 4)	Total score divided by the number of items (no cutoff point). Evaluation benchmark. Analysis of responses separately, or dimensions, or the final score.
ECO-CNV^(17)^	Numerical ordinal 5 points (Low to High ability)	Simple summation (5–25). Average calculation: Low (1.0 to 2.3), Medium (2.4–3.7), High (3.8–5.0)
CSSEF^(18-21)^	Descriptive ordinal (Unsatisfactory=1; Satisfactory=2; Excellent=3)	Summation (8–24). Assessment benchmark. Simple summation. 8 competencies. Includes an open-ended question.
Holistic CSSEF^(18,19,21)^	Descriptive ordinal (Unsatisfactory, Satisfactory, Excellent)	Simple sum of the 2 questions. Evaluation benchmark. Includes an open-ended question.
**Non-validated instruments**		
^(3,7,8,12,20,22-33,35-43)^	Likert; Visual Analogue Scale; Descriptive Ordinal; Numerical Ordinal; Dichotomous; Qualitative	They vary depending on the study (there is no standardization).

**Caption:** PSCI = Public Speaking Competency Instrument; PSCR = Public Speaking Competence Rubric; ECO-CNV *= Evaluación de la Competencia Oral Componente No Verbal*; CSSEF = The Competent Speaker Speech Evaluation Form; CSSEF-Holistic = The Competent Speaker Holistic Speech Evaluation Form

## DISCUSSION

The analysis of the 35 studies included in this scoping review revealed a diversity of indicators. They are mostly proposed by the authors or adapted from existing protocols, with a proportionally smaller number of previously validated tools^(4,14-21)^. This finding points to an important methodological gap that reinforces the importance of standardizing assessment indicators^(15,44)^.

The predominance of assessment indicators developed by the researchers can be attributed to two factors: the need to adapt the indicators to cultural specificities and the intention to broaden the evaluative scope, including more assessment dimensions. Public speaking involves various skills, and encompassing them in a single instrument is a challenging task, which hinders its systematization and makes its reproducibility in other scientific studies more complex^(2,4,16,44)^.

Despite the heterogeneous terminology, the studies used common evaluative indicators, regardless of whether they were author-developed or from validated instruments. They converge around four main domains: discourse structure, nonverbal behaviors, vocal expressiveness, and language, also considering supporting resources and presentation time. Discourse structure recurrently includes indicators such as introduction, organization of ideas, clarity, and conclusion, demonstrating concern for coherence and logical organization in public speaking^(7,19-21,29)^. An effective introduction is responsible for capturing the audience's attention, arousing interest, establishing the speaker's credibility, and clearly presenting the communicative purpose of the discourse^(7,19,21,29)^. The organization of ideas refers to the logical structure of the content, which facilitates the understanding of the progression of thought and contributes to the cohesion of the discourse through clear transitions between its parts^(3,20-23)^. Clarity is associated with the use of accessible language, making the message understandable to the target audience^(21,29)^. Finally, the conclusion reiterates the main points and ends the speech with a memorable final message, helping the audience to retain information^(21,29)^.

Nonverbal behaviors also stood out, with a strong incidence of eye contact, gestures, body posture, and facial expressions, highlighting the importance of nonverbal communication for a good public presentation^(5,17,22)^. Gestures, as an important part of nonverbal communication, are recognized as relevant indicators in the evaluation of public speaking. Studies highlight that their appropriate use helps to convey energy, engagement, and clarity in the message, positively influencing the audience's perception^(3,4,24,38)^.

Another commonly evaluated aspect is body posture, as it reflects attitudes such as confidence, control, and professionalism, while withdrawn postures can signal insecurity or lack of preparation^(3,16,21)^. Evidence also indicates that this element impacts the audience's perception of the speaker's involvement and authority^(4,38)^. Similarly, facial expressions, when appropriate and aligned with the verbal content, are associated with more positive evaluations of communicative performance, as they contribute to the clarity of the message and the speaker's credibility^(4,16,21)^. Conversely, rigid, artificial, or disconnected expressions can negatively interfere with the interpretation of the discourse and compromise its effectiveness^(16,21)^.

Regarding vocal expressiveness, the volume and tone of voice, articulation, and rhythm stood out, reflecting the importance of voice in conveying a message^(8,25,40,42)^. Vocal expressiveness comprises various elements that influence clarity, impact, and audience engagement during an oral presentation. Adequate volume allows the speech to be heard clearly and helps maintain audience interest, especially when associated with variations in intensity throughout the speech^(21)^. Rhythm also plays an important role, reflecting fluency and naturalness in delivery. When used with variation, it provides dynamism and avoids monotony^(4)^. Variation in tone of voice helps to highlight ideas and contributes to audience engagement by avoiding monotonous vocal patterns^(21)^. Clear articulation is essential for speech intelligibility and is considered a marker of communicative competence^(19)^. Although indicators such as volume, tone, articulation, and rhythm were the most frequent, other instruments also observed elements such as pauses, speech rate, intonation, resonance, vocal psychodynamics, melodic curve, vocal projection, emphasis, and fundamental frequency. Although less frequently mentioned, they play a central role in constructing communicative expressiveness and deserve greater attention in future assessment models.

Language proficiency was also widely considered, with indicators related to word choice, content adaptation to the audience, argumentation, and speech fluency – important aspects to ensure that the message is understood, persuasive, and appropriate to the communicative context^(2)^. The appropriate choice of words, vocabulary, and grammar promotes clarity and audience engagement, allowing the message to be understood more easily and with greater impact^(21,23,29,31)^. Furthermore, adapting the content to the audience and the context of the presentation helps to establish a more effective connection between the speaker and listeners, making the speech more meaningful and accessible^(14,21,23,29,32)^. Argumentation, in turn, involves the selection of relevant ideas and their logical organization, using examples and analogies to support the communicative purpose of the speech^(16,23,32,43)^. Speech fluency, characterized by continuity, naturalness, and the absence of excessive interruptions or hesitations, reflects the speaker's preparation and mastery of the content^(2,4)^. When integrated, these elements enhance the persuasive potential of the presentation and ensure that the message is appropriate to the communicative context.

Some indicators identified in the analyzed instruments contribute to a broader understanding of public speaking performance. In turn, the overall evaluation of the speech captures a holistic impression of the performance, integrating multiple subjective dimensions of the discourse^(24)^. Indicators such as the emotion conveyed, spontaneity, and delivery point to authenticity, emotional involvement, and naturalness, connecting with the audience and increasing the impact of the message^(3,16)^. The mention of anxiety in front of an audience reveals the influence of internal emotional aspects on performance, which can interfere with fluency and expressiveness, even in well-prepared speakers^(2)^. These elements, although not grouped into recurring domains, contribute to a broader and more sensitive understanding of communicative competence in real public speaking contexts.

Furthermore, the studies used response scales heterogeneously. The 5-point Likert scale, the most recurrent, has been widely used to assess public speaking because it measures important subjective aspects such as clarity, organization, expressiveness, and engagement^(2,4,16,24,26)^. It is an ordinal scale that captures nuances of performance and perception, being applicable in both self-assessments and assessments by observers^(2)^. Studies have demonstrated its usefulness in structured instruments with multiple domains, enabling more precise analyses of communicative performance^(4,16)^. Furthermore, its standardization favors comparison between groups and experimental conditions, contributing to the reproducibility of findings and the methodological quality of studies in the area^(2,24,26)^.

This study has limitations, especially the challenge of grouping similar indicators described differently in the literature. This process may have introduced variations in analysis standardization. On the other hand, it allowed the findings to be structured into clearer and more comparable domains, favoring their systematization. Furthermore, although scoping reviews recommend not restricting languages, the search was limited to English, Portuguese, and Spanish – the languages with the widest academic circulation in the field and in which the team possesses linguistic competence for critical analysis. This choice sought to ensure consistent data selection and interpretation, although it may have reduced the scope of the findings. The search focused on indexed databases, excluding grey literature due to operational constraints, which may have limited the scope of the mapping. Future reviews should include this type of source to obtain more thorough evidence.

## CONCLUSION

This scoping review identified recurring indicators in the evaluation of public speaking: discourse structure (introduction, development, organization, clarity, conclusion), use of supporting resources, language (appropriateness to the audience, argumentation, pronunciation, fluency), nonverbal behaviors (eye contact, gestures, posture, facial expressiveness), and vocal expressiveness (volume, rhythm, tone, articulation, modulation). Several forms of measurement were observed, with the predominant use of the Likert-type scale. Furthermore, the indicators lacked terminological standardization, posing a challenge for their systematization.
