# Supplementary material for: Indicators for evaluating public speaking in adults: a scoping review
Source: Codas. 2026 Apr 27;38(3):e20250234. doi: 10.1590/2317-1782/e20250234en (PMC13143030; doi:10.1590/2317-1782/e20250234en)
Supplement: Translation

# Translation: Indicadores de avaliação da fala em público em indivíduos adultos: uma revisão de escopo

**DOI:** 10.1590/2317-1782/e20250234pt

**Authors:** Willian Hote Scanferla, Anna Carolina Ferreira Marinho, Adriane Mesquita de Medeiros, Leticia Caldas Teixeira

**Affiliations:** 1 Programa de Pós-graduação em Ciências Fonoaudiológicas, Universidade Federal de Minas Gerais – UFMG - Belo Horizonte (MG), Brasil.; 2 Departamento de Fonoaudiologia, Faculdade de Medicina, Universidade Federal de Minas Gerais – UFMG - Belo Horizonte (MG), Brasil.

**Keywords:** Comunicação, Falar em Público, Comunicação Verbal, Comunicação Não Verbal, Voz

## Abstract

**Objetivo:**

Mapear os indicadores utilizados para observar a fala em público de adultos e identificar as formas de mensuração.

**Estratégia de pesquisa:**

O acrônimo PCC (População, Conceito e Contexto) e descritores combinados foram utilizados para busca nas bases de dados LILACS (BVS), MEDLINE (PubMed), Scopus (Elsevier), Web of Science (Clarivate) e Cochrane.

**Critérios de seleção:**

Foram incluídos estudos sobre a avaliação da fala em público de adultos, conduzida por meio de observação de avaliadores/juízes, publicados em inglês, português ou espanhol, sem restrição quanto ao ano de publicação ou delineamento metodológico. Foram excluídos estudos com indivíduos que gaguejam ou com alterações neurológicas, que utilizassem avaliação tecnológica ou não humana, focados no ensino de língua estrangeira ou baseados apenas na autopercepção.

**Análise dos dados:**

Realizou-se triagem e leitura completa dos estudos, gerenciados no Rayyan, com extração de dados (base de dados, título, autores, ano, idioma, objetivo, método de avaliação, indicadores de análise da fala em público, tipo de escala de resposta e evidências de validade), organizados em planilha para análise e síntese.

**Resultados:**

Foram incluídos 35 estudos publicados entre 2002 e 2024, com concentração em 2016 e predominância de artigos das bases Scopus e Web of Science. Os indicadores identificados foram, em sua maioria, elaborados pelos próprios autores ou adaptados de protocolos existentes. Eles se distribuíram em grandes domínios: estrutura do discurso, uso da linguagem, material de apoio, expressividade vocal e comportamentos não verbais. A escala de mensuração mais utilizada foi a do tipo Likert.

**Conclusão:**

O mapeamento identificou indicadores recorrentes na avaliação da fala em público: estrutura do discurso (introdução, desenvolvimento, organização, clareza, conclusão), recursos de apoio, linguagem (adequação ao público, argumentação, pronúncia, fluência), comportamentos não verbais (contato visual, gestos, postura, expressividade facial) e expressividade vocal (volume, ritmo, tom, articulação, modulação). A forma de mensuração predominante foi a escala do tipo Likert.

## INTRODUÇÃO

Falar em público é definido como a capacidade de realizar uma comunicação oral estruturada e intencional diante de um grupo, com o objetivo de informar, influenciar ou entreter a audiência^(1,2)^. Essa habilidade, considerada desafiadora, é fundamental em diversos âmbitos profissionais e acadêmicos e é considerada um diferencial para o sucesso pessoal e profissional, atendendo às exigências do mercado de trabalho que busca profissionais qualificados e multifuncionais^(2-4)^. Em virtude de sua importância, muitas pessoas se dedicam a aprimorá-la^(1,2,4)^.

Sabe-se que em uma comunicação em público, utiliza-se recursos verbais – que envolvem a mensagem, o uso de palavras e a elaboração do discurso –, recursos não verbais – que englobam a linguagem corporal e os signos visuais de apoio –, e recursos vocais, relacionados à dinâmica vocal, como *pitch*, *loudness*, entonação, acentuação, modulação, pausas, ritmo e velocidade de fala^(3,5,6)^. Além destes, outras dimensões também influenciam a eficiência da apresentação, como a interação com a audiência, a confiança do orador e o impacto geral do discurso, sendo esses fatores frequentemente analisados em avaliações da performance comunicativa^(2,4,5)^.

Estudos sobre treinamento em comunicação oral frequentemente selecionam indicadores para avaliação de juízes, referentes a aspectos comunicativos, antes e após intervenção ou assessoramentos ^(3,7,8)^. Contudo, não há consenso na literatura científica quanto à seleção desses indicadores observáveis, seja em instrumentos validados ou em protocolos sem validação formal. Autores apontam que a ausência de uniformidade evidencia a diversidade de critérios adotados pelos pesquisadores e reforça a necessidade de maiores esforços na construção de parâmetros comuns que favoreçam a prática e a comparabilidade entre estudos, contribuindo para a consolidação do campo ^(2,8,9)^.

Diante dessa lacuna, e considerando exclusivamente a avaliação observável conduzida por avaliadores/juízes, torna-se relevante identificar os indicadores empregados e as formas de mensuração adotadas, a fim de oferecer um panorama atualizado que subsidie a prática avaliativa e possibilite a análise das habilidades comunicativas manifestadas durante a fala em público.

Nesse contexto, as revisões de escopo assumem papel fundamental ao mapear as evidências disponíveis, identificar lacunas de pesquisa e orientar novos estudos e práticas^(10,11)^. Tal enfoque possibilitará reconhecer os indicadores mais convergentes entre os estudos para avaliação das habilidades que necessitam aprimoramento dos oradores em futuras avaliações da fala em público^(2,12)^.

Nesse sentido, o objetivo deste estudo foi mapear os indicadores utilizados para observar a fala em público de adultos e identificar as formas de mensuração.

## MÉTODO

### Estratégia de pesquisa

Trata-se de uma revisão de escopo, elaborada seguindo as diretrizes do *Joanna Briggs Institute* (JBI) para revisões de escopo^(11)^, conforme descrito no PRISMA *Extension for Scoping Reviews* (PRISMA-ScR). O protocolo da presente revisão de escopo foi registrado na *Open Science Framework* (doi:10.17605/OSF.IO/7VKR4).

O acrônimo PCC (P – população, C – conceito e C – contexto) foi utilizado para delineamento do estudo, considerando: P – indivíduos adultos; C – avaliação da fala em público por fonoaudiólogos e instrutores de comunicação e C – na comunicação em público e em estudos realizados em ambientes clínicos ou de treinamento. A pergunta norteadora que motivou esta pesquisa foi: *Quais indicadores são utilizados para observar a fala em público de adultos, como são mensurados e quais convergências se destacam entre eles?*

### Busca

A busca foi realizada de forma eletrônica. As bases de dados utilizadas foram *LILACS (BVS), MEDLINE (PubMed), Scopus (Elsevier), Web of Science (Clarivate) e Cochrane*. As estratégias de busca foram elaboradas com base nos unitermos indexados do *Medical Subject Headings (MeSH)*, Descritores em Ciência da Saúde (DeCS) e descritores livres relacionados ao PCC, foram adaptados para cada plataforma de dados, considerando-se as variações a combinação com os operadores booleanos AND e OR, para obtenção das estratégias finais. A Tabela 1 apresenta a estratégia inicial da busca utilizada nas pesquisas nas bases de dados.

**Tabela 1:** Estratégia inicial de busca nas bases de dados

**Base de Dados**	**Estratégia**
*LILACS (BVS)*	(tw: "Comunicação Verbal" OR "Discurso Público" OR "Fala Pública" OR "Falar em Público" OR oratória OR "Public Speaking" OR "Hablar en Público" OR oratoria OR "Discurso Público" OR "Hablar en Público" OR "Comunicação não Verbal" OR "Nonverbal Communication" OR "Comunicación no Verbal") AND ("Instrumento de Avaliação" OR "Escala de Avaliação" OR avaliação OR escala OR instrumentos OR indicadores OR "Assessment Instrument" OR "Assessment Scale" OR evaluation OR assessment OR scale OR instruments OR indicators OR "Instrumento de Evaluación" OR "Escala de evaluación" OR evaluación) AND ( db:("LILACS"))
*PubMed/Medline*	(("Public Speaking" OR "Verbal Communication" OR "Non-Verbal Communication")) AND (("Assessment Instrument"[Title/Abstract] OR "Assessment Scale"[Title/Abstract] OR Evaluation[Title/Abstract] OR Assessment[Title/Abstract] OR Scale[Title/Abstract] OR Instruments[Title/Abstract] OR Indicators[Title/Abstract]))
*Scopus (Elsevier)*	("Public Speaking" OR Oratory) AND ("Assessment Instrument" OR "Assessment Scale" OR Evaluation OR Assessment OR Scale OR Instruments OR Indicators)
*Web of Science (Clarivate)*	("Public Speaking" OR "Verbal Communication" OR "Non-Verbal Communication") AND ("Assessment Instrument" OR "Assessment Scale" OR Evaluation OR Assessment OR Scale OR Instruments OR Indicators)
*Cochrane*	("Public Speaking" OR "Verbal Communication" OR "Non-Verbal Communication") AND ("Assessment Instrument" OR "Assessment Scale" OR Evaluation OR Assessment OR Scale OR Instruments OR Indicators)

### Critérios de seleção

As etapas de busca, seleção e extração de dados foram conduzidas por dois revisores de forma independente, com um terceiro revisor encarregado de resolver eventuais divergências. As buscas foram realizadas entre julho e setembro de 2024, e não incluíram a literatura cinzenta, como teses, dissertações, trabalhos apresentados em eventos e documentos institucionais, restringindo-se a periódicos indexados.

Foram incluídos estudos sobre a avaliação da fala em público de adultos, conduzida por meio de observação realizada por avaliadores/juízes, publicados em inglês, português ou espanhol, sem restrição quanto ao ano de publicação ou ao delineamento metodológico. A restrição de idiomas foi definida considerando a viabilidade de análise e a garantia de precisão na interpretação dos achados pela equipe.

Foram excluídos estudos com indivíduos que gaguejam ou alterações neurológicas, que utilizassem avaliação tecnológica ou não humana, que tivessem foco no ensino de língua estrangeira ou que se baseassem apenas na autopercepção. A exclusão da autopercepção foi intencional, uma vez que o objetivo desta revisão de escopo foi mapear indicadores observados e julgados por avaliadores/juízes.

### Análise dos dados

A seleção dos estudos foi realizada em duas etapas: inicialmente, foram analisados os títulos, resumos e palavras-chave dos artigos para inclusão; em seguida, procedeu-se à leitura completa dos textos para a aplicação dos critérios de exclusão. Esse processo ocorreu entre agosto e setembro de 2024. Para o gerenciamento das referências, utilizou-se a plataforma *on-line Rayyan* QCRI^(13)^, na qual estudos duplicados foram removidos.

Os dados extraídos abrangeram detalhes específicos do PCC, além dos principais achados sobre os parâmetros utilizados na avaliação da fala em público de indivíduos adultos nos estudos analisados. As informações coletadas incluíram: base de dados, título, autores, ano de publicação, idioma, objetivo do estudo, método de avaliação (questionário próprio ou validado, com identificação do instrumento), indicadores utilizados na análise da fala em público, tipo de escala de resposta (escala do tipo *Likert*, ordinal numérica e ordinal descritiva, qualitativa e quantitativa) e evidências de validade. Os resultados extraídos foram organizados em uma planilha do Excel previamente elaborada para facilitar a análise e a síntese dos dados.

## RESULTADOS

A busca inicial resultou em 3.606 artigos, identificados em diferentes bases de dados, destacando-se *SCOPUS* e *Web of Science*, além da busca manual. Após aplicação dos critérios de elegibilidade, 26 estudos foram selecionados para análise. Não houve divergência entre os dois revisores responsáveis pela triagem, o que dispensou a necessidade de um terceiro avaliador. Adicionalmente, foram incluídos nove estudos identificados por meio da busca manual nas referências dos artigos selecionados, totalizando 35 estudos incluídos nesta revisão (Figura 1). Os artigos finais foram publicados entre 2002 e 2024, com maior concentração em 2016, ano em que cinco estudos foram publicados.

**Figura 1:** Fluxograma de busca e seleção dos estudos que entraram na pesquisa

**Legenda:** n = número de estudos

Entre os indicadores identificados, a maioria 26 (74,3%) foi desenvolvida pelos próprios autores, enquanto nove (25,7%) corresponderam a protocolos validados.

Na Tabela 2, observa-se que o protocolo mais utilizado em quatro estudos foi o *The Competent Speaker Speech Evaluation Form* (CSSEF) de oito itens, com escala de resposta ordinal descritiva e uma questão aberta. A versão holística do CSSEF apresenta dois itens com escala de resposta ordinal e descritiva e foi utilizada em três estudos. O *Public Speaking Competency Instrument* (PSCI) tem 20 itens, escala de resposta do tipo *Likert* e foi utilizada em dois estudos. O *Public Speaking Competence Rubric* (PSCR) apresenta 11 itens, com escala de resposta do tipo *Likert* e foi encontrado dois estudos que utilizaram o instrumento. A Escala de *Evaluación de la Competencia Oral Componente No Verbal* (ECO-CNV) foi encontrada em um estudo desta revisão, tem cinco itens e sua mensuração é feita por escala ordinal numérica. Os protocolos apresentam uma diversidade de indicadores, alguns agrupados por grandes domínios, outros avaliados de forma isolada. A maioria dos instrumentos avaliam a estrutura do discurso, contemplando a introdução, corpo, organização e conclusão do discurso, com subdivisões específicas que contemplam, clareza, objetividade. Os recursos de apoio são recorrentemente avaliados nos instrumentos, embora com nomenclaturas diferentes. Apenas um instrumento não contemplou esse indicador.

**Tabela 2:** Matriz de análise dos instrumentos validados de fala em público

**Instrumento**	**Nº de itens**	**Domínios**	**Indicadores**	**Estudos**
PSCI	20	Introdução	Capacidade de chamar atenção. Propósito claro. Identifica-se a introdução	^(4,14)^
		Corpo	Pontos principais e organização do discurso. Material de apoio	
		Conclusão	Identifica-se a conclusão e seu propósito. Fechamento de pontos. Conclusão forte	
		Entrega	Velocidade de fala. Volume da voz. Gestos. Contato visual. Postura corporal. Expressividade vocal e corporal	
		Competência global	Descritiva	
PSCR	11	Não há domínios avaliam-se os itens/indicadores	Adequação do tópico ao público. Introdução que orienta a audiência. Organização do discurso. Material de apoio convincente. Conclusão reforçada. Escolha de palavras. Expressividade vocal (volume, tom, ritmo). Comportamentos não verbais (postura, gestos, expressões, contato visual); Adaptação ao público. Recurso visual utilizado com habilidade. Argumentação (persuasão, evidências, credibilidade)	^(15,16)^
ECO-CNV	5	Comunicação não verbal	Expressão corporal; Expressão facial; Contato visual; Fluidez; Volume	^(17)^
CSSEF	8	Competências de fala em público	Escolha e delimitação do tema. Clareza da tese. Material de apoio. Padrão organizacional. Linguagem apropriada. Variedade vocal (ritmo, tom, intensidade); Pronúncia/gramática/articulação. Comportamentos físicos (postura, gestos, movimentos, expressões, contato visual, vestimenta). Comentário geral	^(18-21)^
CSSEF Holístico	2	Preparação e conteúdo; Apresentação e entrega	Escolha/delimitação do tema. Comunicação do propósito. Material de apoio. Padrão organizacional. Linguagem apropriada. Variedade vocal. Pronúncia/articulação. Comportamentos físicos não verbais. Comentário geral	^(18,19,21)^

**Legenda:** *PSCI = Public Speaking Competency Instrument; PSCR = Public Speaking Competence Rubric; ECO-CNV = Evaluación de la Competencia Oral Componente No Verbal; CSSEF = The Competent Speaker Speech Evaluation Form; CSSEF-Holistic = The Competent Speaker Holistic Speech Evaluation Form*

A avaliação da linguagem está presente nos instrumentos, com ao menos um indicador contemplado em cada um deles, incluindo aspectos como escolha cuidadosa de palavras, adaptação ao público, argumentação (como persuasão ou credibilidade), boa pronúncia, fluidez do discurso (como pausas e interjeições).

Os comportamentos não verbais, como gestos, contato visual, postura corporal, expressividade corporal, expressões faciais são contempladas em todos os instrumentos analisados.

A maioria dos instrumentos analisados contempla indicadores relacionados à expressividade vocal, especialmente articulação das palavras, velocidade de fala, volume, tom de voz, plasticidade vocal e ritmo da fala.

Em dois instrumentos, o avaliador pode registrar uma observação qualitativa ao final da avaliação, enquanto apenas um deles incluiu um julgamento quantitativo da competência global.

Na Tabela 3, visualiza-se descrição dos indicadores dos 26 estudos que utilizaram instrumentos não-validados, com as respectivas categorias de resposta. Observa-se uma recorrência de indicadores focados em aspectos estruturais do discurso, como introdução (n=3; 11,5%), organização (n=10; 38,5%), clareza de ideias (n=4; 15,4%) e conclusão (n=1; 3,8%). Quanto aos comportamentos não verbais, destacaram-se contato visual (n=17; 65,4%), gestos (n=14; 53,8%), postura corporal (n=11; 42,3%) e expressões faciais (n=8; 30,8%). No domínio da linguagem, a adequação ao público por meio da linguagem foi contemplada em seis estudos (n=6; 23,1%). No domínio da expressividade vocal, os indicadores mais frequentes foram volume (n=14; 53,8%), ritmo (n=4; 15,4%), tom (n=8; 30,8%) e articulação (n=8; 30,8%). Outros aspectos observados incluíram a qualidade do tema (n=1; 3,8%) e o uso de materiais de apoio (n=5; 19,2%). Quanto às escalas de resposta (Tabela 4), os instrumentos utilizam diferentes formatos, incluindo escalas do tipo *Likert* (n=11; 42,3%), numéricas (n=8; 30,8%), ordinais descritivas (n=5; 19,2%), dicotômica (n=5; 19,2%) e visual analógica (n=2; 7,7%) com escores simples ou médias ponderadas. Alguns instrumentos permitem ao avaliador registrar uma observação qualitativa (n=7; 26,9%) e quantitativa (n=2; 7,7%) ao final da avaliação.

**Tabela 3:** Matriz de análise dos instrumentos não validados de fala em público

**INDICADOR**	**ESTUDOS**
Avaliação global	^(12,22-28)^
Persuasão	^(7,12,29,30)^
Confiança	^(8,12,22-24,26-28,30,31)^
Emoção	^(8,29)^
Interação	^(7,12,23,29,30,32)^
Espontaneidade/Entrega	^(3,27,33)^
Organização do discurso	^(3,7,8,22-24,28,29,34,35)^
Introdução	^(7,29,35)^
Clareza de ideias	^(26,27,29,35)^
Corpo do discurso/ Conteúdo da fala	^(33,35-37)^
Conclusão	^(29)^
Qualidade do Tema	^(29)^
Linguagem	^(7,23,29,31,32,36)^
Interjeições	^(7,8,22,24,26-29,31,34,38,39)^
Ansiedade	^(3)^
Apresentação fluida	^(8,36)^
Conteúdo negativo	^(34)^
Argumentação	^(23,27,32,35)^
Psicodinâmica vocal	^(40)^
Expressividade vocal	^(29)^
Qualidade vocal	^(8,41)^
Ressonância	^(8,25,42)^
Tom de voz/*Pitch*	^(3,8,25,27,30,41-43)^
Velocidade de fala	^(3,8,25,34,37,40,41)^
Pausa	^(8,23-25,27,31,34,37,40,42,43)^
Melodia/curva melódica	^(25,37,40)^
Entonação	^(7,22,24,26,28,29,31,32,35,36,38-40)^
Ritmo	^(7,29,32,35)^
Intensidade/Volume/*Loudness*	^(3,7,8,23,25,27,29,34,37-39,41-43)^
Articulação	^(8,23,25,30,37,40-42)^
Projeção vocal	^(35)^
Análise Acústica	^(42)^
Ênfase	^(8,25,37,40)^
Gestos	^(3,8,22-29,33,35,37,38,43)^
Expressividade Corporal	^(8,22,24,26,28,33,34,37-39,43)^
Expessões faciais	^(3,8,25,27,29,35,39,43)^
Postura corporal	^(7,22-27,29,34,35,43)^
Contato Visual/ Contato de olhos	^(7,8,22-24,26,28,29,31,32,34-39,43)^
Movimentos de cabeça	^(25,33)^
Materiais de apoio	^(7,23,27,29,32,35,36)^
Tempo da apresentação	^(7,31,32,35)^
Vestuário	^(23,36,37)^

**Tabela 4:** Descrição das categorias de resposta e métodos de análise dos instrumentos validados e não validados identificados nos estudos

**Instrumentos validados**	**Tipo de escala / Categorias de resposta**	**Método de análise / Escore**
PSCI^(4,14)^	Likert 5 pontos (Excelente=5 a Pobre=1)	Somatório simples (20–100 pontos)
PSCR^(15,16)^	Likert 5 pontos (Deficiente=0 a Avançado=4)	Somatório de notas dividido pelo número de itens (sem ponto de corte). Balizador de avaliação. Analise das respostas separadamente ou dimensões ou escore final
ECO-CNV^(17)^	Ordinal numérica 5 pontos (Baixa a Alta habilidade)	Somatório simples (5–25). Calculo da média: Baixa (1,0 a 2,3) Média (2,4–3,7) Alta (3,8–5,0)
CSSEF^(18-21)^	Ordinal descritiva (Insatisfatório=1; Satisfatório=2; Excelente=3)	Somatório (8–24). Balizador de avaliação. Somatório simples. 8 competências. Inclui questão aberta
CSSEF Holístico^(18,19,21)^	Ordinal descritiva (Insatisfatório, Satisfatório, Excelente)	Somatório simples das 2 questões. Balizador de avaliação. Inclui questão aberta
**Instrumentos não validados**		
^(3,7,8,12,20,22-33,35-43)^	Likert; Visual Analógica; Ordinal Descritiva; Ordinal Numérica; Dicotômica; Qualitativa	Variam conforme estudo (não há padronização)

**Legenda:** *PSCI = Public Speaking Competency Instrument; PSCR = Public Speaking Competence Rubric; ECO-CNV = Evaluación de la Competencia Oral Componente No Verbal; CSSEF = The Competent Speaker Speech Evaluation Form; CSSEF-Holistic = The Competent Speaker Holistic Speech Evaluation Form*

## DISCUSSÃO

A análise dos 35 estudos incluídos nessa revisão de escopo revelou uma diversidade de indicadores. Observa-se que, majoritariamente, eles são propostos pelos próprios autores ou adaptados de protocolos existentes, sendo proporcionalmente menor a presença de ferramentas previamente validadas^(4,14-21)^. Essa constatação aponta para uma lacuna metodológica importante que reforça a importância de padronização dos indicadores de avaliação^(15,44)^.

A predominância de indicadores de avaliação elaborados pelos próprios pesquisadores pode ser atribuída a dois fatores: necessidade de adaptação dos indicadores às especificidades culturais e intenção de ampliar o escopo avaliativo, incluindo mais dimensões de avaliação. Como a fala em público é uma habilidade que envolve várias competências, contemplá-las em um único instrumento torna-se um desafio, o que dificulta sua sistematização e torna mais complexa sua reprodutibilidade em outras pesquisas científicas^(2,4,16,44)^.

Apesar da heterogeneidade nas terminologias utilizadas, existem indicadores avaliativos comuns, independentemente de estarem em instrumentos validados ou construídos pelos próprios autores. Observa-se uma convergência em torno de quatro domínios principais: estrutura do discurso, comportamentos não verbais, expressividade vocal e linguagem. O uso de recursos de apoio e tempo de apresentação também são contemplados. No domínio da estrutura do discurso, observou-se a presença recorrente de indicadores como introdução, organização das ideias, clareza e conclusão, elementos que demonstram preocupação com a coerência e organização lógica na fala em público^(7,19-21,29)^. A introdução eficaz é responsável por captar a atenção do público, despertar o interesse, estabelecer a credibilidade do orador e apresentar claramente o propósito comunicativo do discurso^(7,19,21,29)^. A organização das ideias refere-se à estrutura lógica do conteúdo, que facilita a compreensão da progressão do pensamento e contribui para a coesão do discurso por meio de transições claras entre suas partes^(3,20-23)^. A clareza está associada ao uso de uma linguagem acessível, tornando a mensagem compreensível para o público-alvo^(21,29)^. Por fim, a conclusão retoma os pontos principais apresentados e encerra a fala com uma mensagem final marcante, favorecendo a retenção da informação pela audiência^(21,29)^.

Os comportamentos não verbais também se destacaram, com forte incidência para contato visual, gestos, postura corporal e expressões faciais, evidenciando a importância da comunicação não verbal para uma boa apresentação em público^(5,17,22)^. Os gestos, como parte importante da comunicação não verbal, são reconhecidos como indicadores relevantes na avaliação da fala em público. Estudos destacam que seu uso apropriado contribui para demonstrar energia, engajamento e clareza na transmissão da mensagem, influenciando positivamente a percepção do público^(3,4,24,38)^.

Outro aspecto comumente avaliado é a postura corporal, por refletir atitudes como confiança, controle e profissionalismo, enquanto posturas retraídas podem sinalizar insegurança ou despreparo^(3,16,21)^. Evidências também apontam que esse elemento impacta a percepção da audiência quanto ao envolvimento e à autoridade do orador^(4,38)^. No mesmo sentido, as expressões faciais quando adequadas e alinhadas com o conteúdo verbal são associadas a avaliações mais positivas do desempenho comunicativo, por contribuírem para a clareza da mensagem e a credibilidade do orador^(4,16,21)^. Em contrapartida, expressões rígidas, artificiais ou desconectadas da fala podem interferir negativamente na interpretação da apresentação e comprometer a eficácia da performance^(16,21)^.

Quanto à expressividade vocal, destacaram-se volume e tom da voz, articulação e ritmo, refletindo a valorização da voz na transmissão da mensagem^(8,25,40,42)^. A expressividade vocal é composta por diversos elementos que influenciam a clareza, o impacto e o envolvimento do público durante a apresentação oral. O volume adequado permite que o discurso seja ouvido com nitidez e contribui para manter o interesse da audiência, especialmente quando associado à variação de intensidade ao longo da fala^(21)^. O ritmo também exerce papel importante, refletindo fluência e naturalidade na entrega. Quando utilizado com variação, confere dinamismo e evita a monotonia^(4)^. A variação do tom da voz favorece a valorização de ideias e contribui para o engajamento da audiência, ao evitar padrões vocais monótonos^(21)^. A articulação clara é essencial para a inteligibilidade da fala, sendo considerada um marcador de competência comunicativa^(19)^. Embora os indicadores como volume, tom, articulação e ritmo tenham sido os mais frequentes, outros elementos como pausa, velocidade de fala, entonação, ressonância, psicodinâmica vocal, curva melódica, projeção vocal, ênfase e frequência fundamental também foram observados em outros instrumentos. Esses elementos, ainda que menos citados, têm papel central na construção da expressividade comunicativa e merecem maior atenção em futuros modelos avaliativos.

O domínio da linguagem também foi amplamente considerado, com indicadores relacionados à escolha de palavras, adaptação do conteúdo ao público, argumentação e fluência da fala, aspectos importantes para garantir que a mensagem seja compreendida, persuasiva e apropriada ao contexto comunicativo^(2)^. A escolha adequada das palavras, do vocabulário e da gramática favorece a clareza e o envolvimento da audiência, permitindo que a mensagem seja compreendida com maior facilidade e impacto^(21,23,29,31)^. Além disso, a adaptação do conteúdo ao público e ao contexto da apresentação contribui para estabelecer uma conexão mais eficaz entre orador e ouvintes, tornando o discurso mais significativo e acessível^(14,21,23,29,32)^. A argumentação, por sua vez, envolve a seleção de ideias pertinentes e sua organização lógica, com o uso de exemplos e analogias que sustentam o propósito comunicativo do discurso^(16,23,32,43)^. A fluência da fala, caracterizada pela continuidade, naturalidade e ausência de interrupções ou hesitações excessivas, reflete o preparo do orador e seu domínio do conteúdo^(2,4)^. Quando integrados, esses elementos ampliam o potencial persuasivo da apresentação e asseguram que a mensagem seja apropriada ao contexto comunicativo.

Alguns indicadores identificados nos instrumentos analisados contribuem para uma compreensão ampliada do desempenho em fala pública. A avaliação global da apresentação, por sua vez, permite capturar uma impressão holística da performance, integrando múltiplas dimensões subjetivas do discurso^(24)^. Indicadores como emoção transmitida e espontaneidade ou entrega sinalizam autenticidade, envolvimento emocional e naturalidade, fatores que favorecem a conexão com a audiência e aumentam o impacto da mensagem^(3,16)^. Já a menção à ansiedade diante do público revela a influência de aspectos emocionais internos sobre a performance, que podem interferir na fluência e na expressividade, mesmo em oradores bem preparados^(2)^. Esses elementos, ainda que não agrupados em domínios recorrentes, contribuem para uma compreensão mais ampla e sensível da competência comunicativa em contextos reais de fala pública.

Soma-se a esses aspectos, uma heterogeneidade das escalas de resposta utilizadas, sendo que a escala do tipo *Likert* de cinco pontos foi a mais utilizada. Sabe-se que essa escala tem sido amplamente empregada na avaliação da fala em público por permitir a mensuração de aspectos subjetivos importantes, como clareza, organização, expressividade e engajamento^(2,4,16,24,26)^. Trata-se de uma escala ordinal que permite captar nuances de desempenho e percepção, sendo aplicável tanto em autoavaliações quanto em avaliações por observadores^(2)^. Estudos demonstraram sua utilidade em instrumentos estruturados com múltiplos domínios, possibilitando análises mais precisas do desempenho comunicativo^(4,16)^. Além disso, sua padronização favorece a comparação entre grupos e condições experimentais, contribuindo para a reprodutibilidade dos achados e a qualidade metodológica dos estudos na área^(2,24,26)^.

Este estudo apresenta limitações, entre elas, destaca-se o desafio de agrupar indicadores semelhantes descritos de formas distintas na literatura. Esse processo pode ter introduzido variações na padronização da análise; por outro lado, permitiu estruturar os achados em domínios mais claros e comparáveis, favorecendo sua sistematização. Além disso, embora em revisões de escopo se recomende não restringir idiomas, a busca foi limitada ao inglês, português e espanhol, línguas de maior circulação acadêmica na área e nas quais a equipe possui competência linguística para análise crítica. Essa escolha buscou assegurar consistência na seleção e interpretação dos dados, ainda que possa ter reduzido a abrangência dos achados. A busca concentrou-se em bases indexadas, sem incluir literatura cinzenta, essa decisão, motivada por restrições operacionais, pode ter limitado a abrangência do mapeamento. Futuras revisões devem contemplar esse tipo de fonte para ampliar a completude das evidências.

## CONCLUSÃO

O mapeamento desta revisão de escopo identificou indicadores recorrentes na avaliação da fala em público: estrutura do discurso (introdução, desenvolvimento, organização, clareza, conclusão), uso de recursos de apoio, linguagem (adequação ao público, argumentação, pronúncia, fluência), comportamentos não verbais (contato visual, gestos, postura, expressividade facial) e expressividade vocal (volume, ritmo, tom, articulação, modulação). Observaram-se várias formas de mensuração, com destaque para o uso predominante da escala do tipo *Likert*. Constatou-se, ainda, ausência de padronização terminológica entre os indicadores o que representa um desafio para sua sistematização.
